# Symptoms, Mechanisms, and Treatments of Video Game Addiction

**DOI:** 10.7759/cureus.36957

**Published:** 2023-03-31

**Authors:** Shabina Mohammad, Raghad A Jan, Saba L Alsaedi

**Affiliations:** 1 Department of Anatomy, Taibah University, Madinah, SAU; 2 Collage of Medicine, Taibah University, Madinah, SAU

**Keywords:** treatment of addiction, addiction, internet gaming disorders, mechanism of addiction, video game addiction

## Abstract

Video game addiction is defined as the steady and repetitive use of the Internet to play games frequently with different gamers, potentially leading to negative consequences in many aspects of life. As recent technological development has given easy access to gaming on many devices, video game addiction has become a serious public health issue with increased prevalence. Many studies have shown that video game addiction leads to changes in the brain that are similar to those that occur in substance addiction and gambling. Evidence has also shown that there is an association between video game addiction and depression, as well as other psychological and social problems. In light of these issues, our review article aims to increase awareness of video game addiction in society. The main objectives of this review are as follows: to describe the mechanism of addiction, to consider whether video game addiction is a real addiction, and to highlight the signs and symptoms of addiction. In addition, we identify the consequences of video game addiction and possible treatments for addicts. The information was extracted from high-quality research papers and reliable websites like PubMed and ScienceDirect.

## Introduction and background

Video game addiction falls into the category of Internet gaming disorders (IGDs), which have been strongly correlated with motivational control issues and are regularly compared with gambling [1]. Many studies have suggested that behavioral addiction can result from compulsive use of the internet [2-4]. Although the spectrum of internet addiction includes video gaming, online shopping, gambling, and social networking, video game addiction seems to be its most studied form [5]. Currently, according to the Diagnostic and Statistical Manual of Mental Disorders, Fifth Edition (DSM-5), video game addiction is defined as the steady and repetitive use of the Internet to play games frequently with different gamers, which leads to clinically significant distress and psychological changes as demonstrated by five or more criteria in a year [1,6].

Evidence has shown that addiction can cause changes in some areas of the brain, such as the prefrontal cortex (PFC), the ventral striatum (VS), and the dorsal striatum (DS) [5]. In 1999, neuroimaging evidence showed that increases in dopamine (DA) levels in the brain are associated with pleasure and euphoria [7,8].

Cross-sectional studies revealed that those with IGD played video games longer, usually missed classes in school, had lower grades, reported more sleep problems, and showed more addiction to video games than those without IGD [1]. Yee suggested that gamers are generally more youthful and may have low self-esteem or emotional disturbances, and those with emotional disturbances might be more vulnerable to addiction to gaming [9]. In video games, gamers can explore different sides of themself. They can be increasingly vocal and experience assuming the role of a leader, among others. Unfortunately, problems can arise when these youthful gamers become dependent upon the personalities they create in online games - blurring the line between reality and the game [10]. The rapid development of technology has given young gamers further access to games, such as advanced mobile phones, tablets, and personal computers (PCs). These online video games usually have tasks and achievements. As an achievement in video games occurs quickly, they are especially alluring for youthful gamers as a method for experiencing fun. Games depend on such feelings, as they often delight the individual and give them satisfaction in their accomplishment. Moreover, the use of a microphone or text chat is enabled in many games, allowing gamers to satisfy their need for relatedness by speaking with others [11]. Gamers regularly experience difficulty with social connections and feel forlorn-lacking a sense of belonging. This inclination can be particularly strong among kids and youths who have never felt like they belong to a place in reality, and most likely their only friends are also gamers [10].

Video game addiction may have both short and long-term impacts on gamers, including various emotional, psychological, and neurological effects. A few studies have demonstrated that anxiety and depression are common among individuals who are dependent on video games [6,12]. In the new generation of children, physical activity time is less and shorter in duration when compared with the parent's generation because children’s activities moved toward indoor more than outdoor play. There is a negative relationship between the time spent on online gaming and exercise and that leads to a sedentary lifestyle which is a risk factor for many medical health conditions such as obesity, diabetes, and coronary artery disease [13]. According to the American Medical Association, approximately 90% of young Americans play computer games. Furthermore, 15% of these gamers (i.e., over five million children) could be considered addicted [10]. Issues arising from gaming have become so serious that the first detox center for video game addiction opened in 2006 in the Netherlands [14]. As described by Keith Bakker, director of Amsterdam-based Smith and Jones Addiction Consultants and their treatment center, although video games may look innocent, they can be as addictive as gambling or drugs and just as difficult of a habit to break [11]. A study in an international school in Buraidah, Saudi Arabia showed that 16% of the 276 students were addicted to video games, and the data showed a strong relationship between video game addiction and psychological distress [6]. In light of these issues, our review article aims to increase awareness of video game addiction in society.

## Review

Mechanism of addiction

Researchers have demonstrated that at the initial phases of the intentional use of any substance, the choice to utilize it is made by the brain, specifically the PFC and the VS, as habituation to utilize and compulsion begins. In the DS, brain activity changes and become progressively activated through dopaminergic innervation [15]. There are also changes in the dopaminergic pathways of the brain, particularly those of the anterior cingulate (AC), the orbitofrontal cortex (OFC), and the nucleus accumbens (NAc), due to the repetitive long-term use of the substance, which may result in a decreased response to natural reward and diminished control over seeking and using the substance [12,16]. The long-term use of substances decreases synaptic activity [17], and the brain becomes progressively responsive by craving substance cues like accessibility [18,19]. In the case of video game addiction, seeing the game or the controller can lead to cravings. Cravings for substance use involve complicated interactions between various brain regions [20]. The OFC plays a significant role in such stimulation-affecting behavior, as does the amygdala (AMG) and the hippocampus (Hipp) [21]. Eventually, an increase in the amount of substance is required to create the desired impact, and the brain’s natural reward system becomes inadequate, prompting the activation of an anti-reward system that diminishes the ability of the addicted to finding biological reinforcers pleasurable [22]. Withdrawal symptoms can also develop, which are due to the absence of DA in the mesocorticolimbic pathways [21]. Research has demonstrated that changes in brain activity generally occur in substance addiction after a compulsive commitment to use [23], which suggests that there may be similar changes associated with video game addiction. However, as mentioned previously, increases in DA levels occur by various mechanisms. In this way, the DA theory of addiction explains the powerful activation of mesolimbic DA, which is a strong feature of every addictive substance. However, although the increase in DA may clarify the intense fortifying impacts of addictive substances, it does not clarify associated long-term behavioral anomalies such as desiring and relapsing, which are obvious features even when DA levels return to normal after the clearance of the substance [24]. Therefore, changes in DA levels cannot be the reason for the adaptive behavior that presents long after the substance has been cleared from the brain [25,26]. Accordingly, it has been proposed that the changes induced by a substance in specific neurotransmission circuits of the mesocorticolimbic area play a role in the pathological behavior of addictive patients [27].

Whether it is real addiction or not

Numerous governments see the compulsive playing of online video games as a serious general medical problem and have set up treatment facilities, particularly in China and South Korea [28]. However, whether video games can lead to a real addiction is still hotly debated, and the question arises as to whether a game can be considered an intoxicant. The neurological proof suggests that video games might act as substances, with convincing similarities between their impacts on brain chemistry [5]. Professor Nancy Petry of the American Psychiatric Association committee considered adding video game addiction to the most recent diagnostic manual but ultimately chose to wait for further consideration [28]. In recent years, the concept of becoming addicted to behavior has grown in popularity, especially with growing neuroimaging evidence of the brain’s response to such behavior. For example, gambling has been shown to activate the brain’s reward system in a way that is similar to that of a substance [28]. In 2011, a functional magnetic resonance imaging (fMRI) study on 154 participating 14-year-olds by Simone Kühn of Ghent University in Belgium found that frequent gamers had increased grey matter in the left VS - a change that may result from increased DA discharge and also appears in those addicted to gambling [29]. According to the WHO, adding video game addiction to the ICD-11 depends on evidence and agreement among specialists from various countries that are associated with the procedures of specialized consultations. The inclusion of video game addiction in the ICD-11 follows the improvement of treatment programs for individuals with similar conditions in many countries, which will bring more attention to the dangers of this addiction among health professionals, as well as to available avoidance and treatment measures. For gaming addiction to be diagnosed, the signs and symptoms must be of adequate seriousness to bring impairment in different aspects of the person’s life, with the effects normally being obvious for at least one year [30].

Sign and symptoms

It is important to be able to identify the signs and symptoms of video game addiction, and the sooner one looks for help, the better the outcome. As video games are still a relatively new technology, therapists may disregard the signs of video game addiction. To be able to make informed choices and act successfully in navigating addiction, the following signs can be used as a guide [10].

Preoccupation with Gaming

Video game addiction starts when gamers become preoccupied with video games, where they will think and fantasize about the game when they are not playing it when they should be focusing on other things, such as schoolwork [10].

Lying or Hiding Gaming Use

Gamers may play games for uncountable numbers of hours and days, leading to a lack of eating, resting, or personal hygiene. They may also lie to loved ones about what they are truly doing for hours in their rooms. Gamers may tell their parents that they are getting their work done, say that they are using their PC for work, or make up many reasons why they cannot go out and socialize [10].

Loss of Interest in Other Activities

As video game addiction increases, the person’s interest in daily activities they used to enjoy decreases [31]. For example, one gamer’s mother said that her child cherished baseball and played varsity on his secondary school team until he discovered Xbox Live, which led to declining grades [32]. However, she only realized that there was a problem when he stopped playing baseball, as he cherished the sport to an extreme.

Social Withdrawal

One’s character might also change as the addiction increases. For example, social people may remove themselves from their loved ones to invest more time in play. Otherwise normal and happy children may be inclined to simply make friendships in the game, which can become more important than those in real life [10].

Psychological Withdrawal

Gaming addicts may experience loss when they cannot play a game, as they miss it and feel the need to play it. This inclination can become intense to the point that they become emotionally unstable when compelled to abandon the game. As a result, they cannot focus on anything else except playing [10].

Defensiveness and Anger

When gaming addicts need to play or when they are compelled to stop playing, they can become defensive and furious. Guardians attempting to set time limits for the game have reported that their children can become angry, unreasonable, and even brutal in response [10].

Gaming Addicts Use the Game World as a Psychological Escape

Video games, as an easy way to mitigate upsetting emotions, can turn into a safe space that is removed from real-world issues [10]. Those who feel shy or alienated from their peers can feel more confident while gaming and this fictional life can become more fulfilling than their real life [32].

Continued Use Despite Its Consequences

Frequently, gamers have the urge to be the best in the game. Therefore, the more they develop and progress in the game, the more they have to play it. This progress is found particularly in journey-type games, where gamers join each other and complete missions in the game together [10]. Ultimately, gaming addicts may continue game use despite its negative effect on their lives. Although young gamers may drop out of school and disregard their hygiene and self-care just to play, and adults may lose their jobs or relationships, they often continue to play [32].

Consequences

Video game addiction can lead to a number of serious consequences. Gamers can forget that they need to sleep and eat, or even how to communicate with people in the real world. Gamers may play for 10, 15, or even 20 hours in a single gaming session. Only a couple more minutes can turn into hours as the gamer progresses to the next challenge. They may also endure various medical issues resulting from back strain, eye strain, and carpel tunnel syndrome. For example, one addicted gamer said that he stopped washing himself, ate very little food, and experienced weight loss [10]. The gamer’s appearance had deteriorated so much that his mom said that he looked like a heroin addict. Although gamers make friends in the game, their friendships or relations in real life can end up being damaged. Furthermore, often it is not only the gamer that suffers the consequences of their addiction. In South Korea, parents were arrested because their four-month-old daughter died due to suffocation after they left her alone in the house for many hours while they played World of Warcraft at a nearby café [33]. In another similar story, parents from Reno were playing video games so obsessively that they forgot about their children - a two-year-old boy and a girl who was almost one year old - who were seriously malnourished and near death when doctors saw them. The parents pleaded guilty to child neglect and faced a 12-year jail sentence. According to the Associated Press, authorities said the parents were so distracted by video games, mainly the fantasy role-playing Dungeons and Dragons series, that they forgot to take care of their children [34].

Treatment

Research on video game addiction treatment is ongoing, only a few clinical trials have been conducted. Some therapists have suggested cognitive behavior therapy (CBT) [35]. As a prevention strategy for IGD, exercises, and physical activities have been recommended. Particularly, outdoor activities and sports [36].

CBT

Based on the peer-reviewed literature, CBT seems to be the most common treatment for video game addiction and IGD [37,38]. Specifically, substance abuse treatment is often applied, including stimulus control, learning proper adapting reactions, self-monitoring strategies, cognitive rebuilding, addiction-related critical thinking, and withdrawal regulation methods with exposure [37,39]. One systematic review and meta‐analysis suggested that this treatment option is effective for IGD as a short‐term intervention for reducing IGD and symptoms of depression. However, the effectiveness of CBT in reducing the amount of time that is spent on games was unclear. Therefore, there is a need for more studies to determine the long‐term benefits of CBT for IGD [40].

PIPATIC

Programa Individualizado Psicoterapéutico para la Adicción a las Tecnologías de la Información y la Comunicación (PIPATIC) has been used to treat addiction to technology. PIPATIC is an individualized psychotherapy program that is available for people aged 12 to 18 years who have IGD and is designed to integrate several areas of intervention. PIPATIC comprises six modules: psychoeducational, treatment as usual, intra-personal, interpersonal, family intervention, and development of a new lifestyle [39]. The program uses many psychotherapeutic strategies, one of them is CBT [41]. It was suggested that the use of many psychotherapeutic strategies is more effective than one (Dong and Potenza 2014; Orzack et al. 2006; Sheketal 2009; Therien et al. 2014). The goals of the program are to reduce the symptoms of video game addiction and to improve the well-being of adolescents, and its initial findings have been encouraging [39]. The program has a duration of six months and involves 22 to 45-minute sessions each week for video game addicts and their families. The PIPATIC intervention is a person-to-person approach that involves a qualified clinical psychologist [41], with therapeutic homework to strengthen the establishment that is related to the therapeutic changes required to help change everyday behavior. In the PIPATIC program, the family collaborates to achieve treatment progress, and it is usually them who ask for therapeutic care or psychological treatment [42]. Although the PIPATIC program appears promising, there are some limitations. First, it was designed to focus on only one specific population, adolescents. Second, it must commit to specific methodological standards for the application and evaluation of the intervention, and it is difficult for addicts to adapt to these particular needs. However, results have suggested that the treatment is effective, as data from 17 video-game addicts that went through the program showed a decrease in time spent playing video games and improvement in many important daily life activities (e.g., family and educational or occupational functioning) [41].

Pharmacotherapy

The medications trial that has been done were on drugs usually used to treat depression or attention deficit hyperactivity disorder (ADHD). Bupropion is one of the medications used for depression. It has been investigated as a treatment for IGD in randomized clinical trials [43,44]. The drug was superior to the control group with no medication [43]. Also, superior compared to the placebo group in decreasing the symptoms of IGD that were maintained for four weeks after the treatment [44]. Another antidepressant is escitalopram. Compared to bupropion it was inferior in reducing the symptoms [43].

## Conclusions

Many studies suggest that the changes that occur in video game addicts are similar to those that occur in other addictions, such as substance-related addiction and gambling. The WHO has added video game addiction to the ICD-11, with adolescents being the most susceptible. Video game addiction can lead to many serious consequences for gamers and the people close to them. Although research on video game addiction treatment is ongoing, only a few clinical trials have been conducted on the efficacy of the treatment options, for example, CBT, PIPATIC, and pharmacotherapy.
